# Bacterial Diversity and Antimicrobial Resistance of Microorganisms Isolated from Teat Cup Liners in Dairy Farms in Shandong Province, China

**DOI:** 10.3390/ani14152167

**Published:** 2024-07-25

**Authors:** Guangwei Yan, Shengnan Wang, Yuehui Cui, Kun Xue, Yongxia Liu, Jianzhu Liu

**Affiliations:** 1 College of Veterinary Medicine, Shandong Agricultural University, Tai’an 271018, China; 17863666615@163.com (G.Y.); wangshengnan8989@163.com (S.W.); yuehuicui90@163.com (Y.C.); xuekun2659664143@163.com (K.X.); 2 The ShangHai Hanvet Bio-Pharm Co., Ltd., Shanghai 200135, China; 3 Research Center for Animal Disease Control Engineering, Shandong Agricultural University, Tai’an 271018, China

**Keywords:** dairy farm, teat cup, bacteria diversity, antimicrobial resistance

## Abstract

**Simple Summary:**

Assessing the quality of raw milk is crucial for humans’ well-being. This study aimed to evaluate the bacterial composition and antimicrobial resistance in the tea cup liners of dairy cow milking equipment. The objective was to determine the potential risk of bacterial contamination in raw milk during the milking process and the transmission of antimicrobial-resistant bacteria through milk sources. The study found that *Bacillus* spp. were the most prevalent bacteria identified on the teat cup liners. A significant proportion of these bacteria showed significant resistance to various antibiotics, such as lincomycin, sulfadiazine, and streptomycin. Multiple antimicrobial resistance genes were also detected, indicating the potential transmission of antimicrobial-resistant bacteria in the milk samples. These findings underscore the imperative to enhance disinfection protocols within dairy farms and milking equipment. The results accentuate the enduring peril of disseminating antimicrobial-resistant bacteria via contaminated milk sources. Taking heed of this finding will contribute to the sustainable development of the dairy industry while mitigating the hazards associated with food safety. Increasing awareness of the potential dissemination of antimicrobial-resistant bacteria through dairy products can foster the implementation of appropriate measures to ensure the secure production and consumption of such products, thereby safeguarding public health and welfare.

**Abstract:**

Global milk consumption exceeds 800 million tons a year and is still growing. Milk quality and its products are critical to human health. A teat cup makes direct contact with the cow’s teats during milking and its cleanliness is very important for the quality of raw milk. In this study, the microorganism from post-milking teat cup liners were collected from six dairy farms in Shandong Province of China, the bacterial species were identified using microbial mass spectrometry, the minimum inhibitory concentrations of the isolated strains against ten antimicrobial agents were determined using the broth microdilution method, and the antimicrobial resistance genes were detected by PCR. The results indicated that the most frequently isolated bacteria in this study were *Bacillus licheniformis* (39/276, 14.13%), followed by *Bacillus pumilus* (20/276, 7.25%), *Bacillus cereus* (17/276, 6.16%), and *Bacillus subtili* (16/276, 5.80%). The isolates exhibited the highest average resistance to lincomycin (87.37%), followed by sulfadiazine (61.05%) and streptomycin (42.63%); the highest detection rate of resistance genes was *Sul*1 (55.43%), followed by *ant*(4’) (51.09%), *tet*(M) (25.36%), *bla*_KPC_ (3.62%) and *qnrS* (3.62%). These findings imply the necessity for enhanced measures in disinfecting cow udders and milking equipment, highlighting the persistently challenging issue of antimicrobial resistance in Shandong Province.

## 1. Introduction

Milk and its products are foods with high nutritional content, providing humans with high-quality proteins, bioavailable amino acids, and a wide range of vitamins and minerals [1]. However, the milking process can lead to the contamination of milk with bacteria, which can seriously affect the quality of the milk and threaten food safety. Although cows’ udders and milking equipment are usually disinfected prior to milking, the effectiveness of disinfection and the presence of pathogenic bacteria residues after disinfection are still not very certain. To assess the probability of bacterial contamination of the milk source through the milking process and the risk of transmission of antimicrobial resistance genes (ARGs) through the milk source, we collected samples from teat cup liners after milking from six dairy farms in the Shandong Province of China and tested the samples for bacterial abundance, bacterial antimicrobial resistance, and the presence of ARGs.

Understanding the bacterial diversity and antimicrobial resistance (AMR) of the teat cups, which are an important part of the milking machine and whose liners come into direct contact with the skin on the udder surface and raw milk during milking, will help assess the quality of raw milk and has implications for food safety as well as public health. Bacteria that come into contact with the lining of the teat cups have a high probability of entering the raw milk, which can lead to milk spoilage and food poisoning. *Bacillus cereus* is a common and prevalent foodborne pathogen in Chinese dairy products, increasing the risk of food poisoning and other illnesses [2]. In addition to this, the spread of antimicrobial-resistant bacteria through the food chain can lead to an increase in acquired antimicrobial resistance, posing a major public health threat [3]. Bacteria such as Staphylococcus aureus, Streptococcus dysgalactiae, and Escherichia coli have been identified as common pathogens that cause mastitis in dairy cows, and these bacteria are resistant to many first-line antimicrobial drugs [4]. Mastitis is one of the most common causes of antimicrobial use in dairy herds to treat cows, and high levels of antimicrobial use can create selection pressure on bacteria, leading to the predominance of antimicrobial-resistant strains. Pathogen resistance in turn can lead to lower cure rates in clinical-type mastitis cases and can be transmitted to humans through the food chain, leading to public health problems [5]. AMR is a global health concern. The discovery of *Klebsiella pneumoniae* carrying the NDM-1 enzyme in 2009 raised serious concerns. NDM-1 can break down many antibiotics, including carbapenems, which are often the last resort for treating resistant infections. This highlights the urgent need to address AMR [6,7]. In 2016, another resistance gene *mcr-1* against colistin resistance was detected, and treating bacterial infections containing colistin resistance genes will also be a major challenge, as antimicrobials against Gram-negative bacteria are very limited [8].

The heavy use of antimicrobials in past decades has put selection pressure on bacteria, making it easier for antimicrobial-resistant strains to survive. This has led to the development of antimicrobial resistance, which poses a major threat to global public health [9]. This study investigated the prevalence of bacteria and antimicrobial resistance in milk cup liners from dairy farms in Shandong Province. The aim was to assess the potential for milk source contamination during milking and the risk of transmitting animal-derived antimicrobial-resistant bacteria to humans via the milk supply. The findings contribute to data for clinical antimicrobial use, milk safety, and the sustainable development of the dairy industry.

## 2. Materials and Methods

### 2.1. Sample Collection

Samples were obtained from Tai’ an (TA), Dongying (DY), and Qingdao (QD) in Shandong Province, and two dairy farms in each region were selected for sampling. The post-milking teat cup liners were randomly selected with sterile latex gloves using autoclaved cotton swabs and immediately placed in 1.5 mL centrifuge tubes containing sterile saline, and the samples were stored in a cooler with ice packs and transported to the laboratory within 24 h for testing. A total of 248 samples were collected from six dairy farms from March to May 2022.

### 2.2. Bacterial Isolation and Identification

The collected samples were all inoculated in LB agar (Solarbio, Beijing, China) containing 5% defibrinated sheep blood and incubated for 24 h at 37 °C in a constant temperature bacterial incubator. After selecting individual colonies and repeating the above incubation steps 3–5 times, a total of 276 bacteria were isolated and identified using a microbial mass spectrometer (MALDI Biotyper, Bruker, Ettlingen, Germany).

### 2.3. Antimicrobial Susceptibility Tests

Antimicrobial resistance characteristics of all isolates were determined using the microbroth dilution method according to Clinical and Laboratory Standards Institute Guidelines 2020 (CLSI, 2020). The following antimicrobial agents were tested: colistin sulfate (PB), ampicillin sodium (AMP), streptomycin sulfate (STS), neomycin sulfate (Nm), lincomycin hydrochloride (LIN), sulfadiazine (SD), ceftriaxone sodium (CRO), gentamicin sulfate (GM), levofloxacin (LVX), and doxycycline hydrochloride (DO). All the above drugs were purchased from the Shanghai MacLean Company (Shanghai, China). *Escherichia coli* ATCC 25922 and *Staphylococcus aureus* ATCC 29213 were used as quality control strains. Refer to CLSI 2020 and the European Commission Antimicrobial Susceptibility Test (https://www.eucast.org/) to assess antimicrobial susceptibility results. Multidrug resistance (MDR) is defined as acquired insusceptibility to at least one of three or more classes of antimicrobials.

### 2.4. Detection of Antimicrobial Resistance Genes

The boiling method for extracting bacterial genomic DNA was optimized based on the original method [10]. PCR amplification of antimicrobial resistance genes was performed with synthetic primers (Sangon Biotech, Shanghai, China) and PCR products were evaluated by agarose gel electrophoresis. Seven classes of antimicrobial resistance genes were examined, including polymyxin resistance genes (*mcr-1*); β-lactam resistance genes (*bla*_KPC_, *bla*_NDM-1_); sulfonamide resistance genes (*sul*1); aminoglycoside resistance genes (*aph(2″)*, *ant(4′)*); quinolone resistance genes (*qnrS*); tetracycline resistance genes (*tet*(M)); and lincosamine resistance genes (*lnuB*) (primer sequences and sources are shown in Supplementary Table S1).

### 2.5. Statistical Analysis

Statistical analysis was performed using a Fisher’s exact test to determine significant differences in the proportions of antibiotic-resistant strains among the various sampling environments. Data organization was completed using Microsoft Excel 2020 (Microsoft Corporation, Redmond, WA, USA), and graphs were generated using Origin 2022 (OriginLab Corporation, Northampton, MA, USA).

## 3. Results

### 3.1. Bacterial Isolation

**Figure 1:**
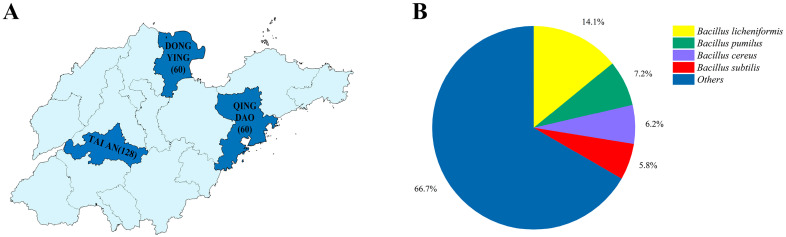
Source of samples and approximate isolation of bacteria. (**A**) Distribution map of 248 samples collected in Shandong Province, with 128 samples collected in TA, 60 samples collected in DY, and 60 samples collected in QD. (**B**) Approximate isolation of bacteria in Shandong. *Bacillus licheniformis* (39/276, 14.13%) was the most frequently isolated, followed by *Bacillus pumilus* (20/276, 7.25%), *Bacillus cereus* (17/276, 6.16%) and *Bacillus subtilis* (16/276, 5.78%). Abbreviations: TA, Tai’an; DY, Dongying; QD, Qingdao.

In this survey, we collected 248 samples from three regions in Shandong Province, including 128 samples from TA, 60 samples from DY, and 60 samples from QD (Figure 1A). Since the milking equipment and udder are disinfected prior to milking, not all samples were isolated for bacteria. The positive rate of bacterial isolation of samples represents, to some extent, the effectiveness of the disinfection procedure and the cleanliness of the udder. Among them, the lowest bacterial isolation rate was 39.47% (30/76) in one farm in Tai’an, and the highest positive rate was 100% (30/30) in one farm in Qingdao, with an average positive rate of 61.69% (153/248), indicating that the probability of bacterial contamination of milk through the milking process was more than 50% (Table 1), with large differences in sanitary conditions and disinfection procedures among different farms. In this study, 276 isolates belonging to 73 species were identified from 248 samples, of which *Bacillus licheniformis* (39/276, 14.13%) was the most frequently isolated, followed by *Bacillus pumilus* (20/276, 7.25%), *Bacillus cereus* (17/276, 6.16%) and *Bacillus subtilis* (16/276, 5.78%) (Figure 1B). *B*. *cereus* is one of the major foodborne pathogens, and ingestion of food containing this bacterium can lead to the risk of foodborne illness, mainly in the form of gastrointestinal symptoms such as diarrhea and vomiting [11].

**Figure 2:**
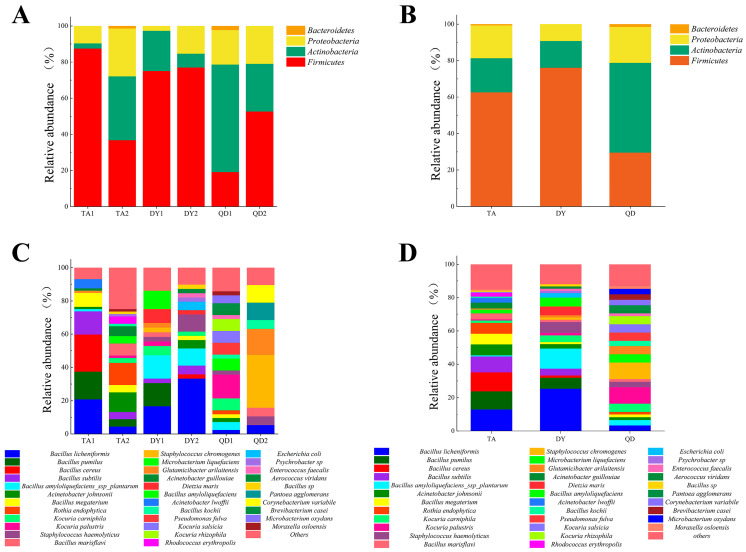
Bacteria isolated from teat cup liners in Shandong. (**A**) Distribution of isolates at the phylum level. The largest proportion in both TA and DY regions was *Firmicutes*, and the largest proportion in QD region was *Actinobacteria*. (**B**) Distribution of isolates at the phylum level. Specifically for each farm, the largest proportion of QD1 was *Actinobacteria* and the largest proportion of all other farms was *Firmicutes*. (**C**) Distribution of isolates at the species level. The largest number of isolates in TA were *Bacillus licheniformis*, *Bacillus cereus*, and *Bacillus pumilus*; the largest number of isolates in DY were *Bacillus licheniformis*, *Bacillus amyloliquefaciens ssp plantarum*, *Staphylococcus haemolyticus*; the largest number of isolates in QD were *Staphylococcus chromogenes*, *Kocuria palustris*, and *Glutamicibacter arilait plantarum*. (**D**) Distribution of isolates at the species level. Specifically for each farm, the highest amount of isolates was *Bacillus cereus* in TA1, *Rothia endophytica* in TA2, *Bacillus licheniformis* in both DY1 and DY2, *Kocuria palustris* in QD1, and *Staphylococcus chromogenes* in QD2. Abbreviations: TA, Tai’an; DY, Dongying; QD, Qingdao; TA1, Taian’s first sampling farm; TA2, the second sampling farm in Tai’an; DY1, Dongying’s first sampling farm; DY2, the second sampling farm in Dongying; QD1, Qingdao’s first sampling farm; QD2, the second sampling farm in Qingdao.

Subsequently, we statistically analyzed the distribution of isolates at the phylum level, and the data showed that *Firmicutes* accounted for the largest proportion in both TA and DY, while *Actinobacteria* accounted for the largest proportion in QD (Figure 2A). Specifically for each farm, *Firmicutes* accounted for the largest share, except for Qingdao’s first sampling farm (QD1), where *actinobacteria* accounted for the largest share (Figure 2B). At the species level, the largest number of isolates in TA were *B. licheniformis*, *B. cereus*, and *B. pumilus*; the largest number of isolates in DY were *B. licheniformis*, *Bacillus amyloliquefaciens ssp plantarum*, *Staphylococcus haemolyticus*; the largest number of isolates in QD were *Staphylococcus chromogenes*, *Kocuria palustris*, and *Glutamicibacter arilaitensis* (Figure 2C). Specifically for each farm, the most isolated species was *B. cereus* in Taian’s first sampling farm (TA1), *Rothia endophytica* in the second sampling farm in Tai’an (TA2), *B. licheniformis* in both Dongying’s first sampling farm (DY1) and the second sampling farm in Dongying (DY2), *Kocuria palustris* in QD1, and *Staphylococcus chromogenes* in the second sampling farm in Qingdao (QD2) (Figure 2D). 

**Figure 3:**
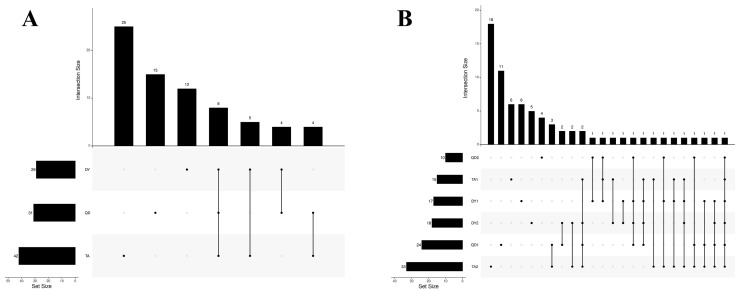
Differences between bacteria isolated from different sample sources. (**A**) Differences between bacteria isolated in the three cities. An amount of 42 species of bacteria were most abundantly isolated in TA, followed by 31 species in QD and 29 species in DY. Among them, 25 species of bacteria were independently detected in TA, 15 species in QD, and 12 species in DY, and eight species of the same bacteria were isolated in the three areas. (**B**) Differences among bacteria isolated from the six farms. TA2 and QD1 had the most species of bacteria independently, 18 and 11 species, respectively, and only one species of bacteria common to the six farms was Bacillus licheniformis. Abbreviations: TA, Tai’an; DY, Dongying; QD, Qingdao; TA1, Taian’s first sampling farm; TA2, the second sampling farm in Tai’an; DY1, Dongying’s first sampling farm; DY2, the second sampling farm in Dongying; QD1, Qingdao’s first sampling farm; QD2, the second sampling farm in Qingdao.

In order to understand the differences of bacterial diversity among each region, the isolated bacteria were analyzed statistically. The results showed that TA isolated the most abundant bacterial species with 42 species, followed by QD with 31 species and DY with 29 species. Among them, 25 species of bacteria were independently detected in TA, 15 species in QD, 12 species in DY, and eight species of the same bacteria were isolated from the three regions (Figure 3A). Specifically for the farms, TA2 and QD1 had the most species of bacteria independently, with 18 and 11 species, respectively, and only one species of bacteria common to the six sites was *B. licheniformis* (Figure 3B).

### 3.2. Antimicrobial Resistance and MDR Profile

**Figure 4:**
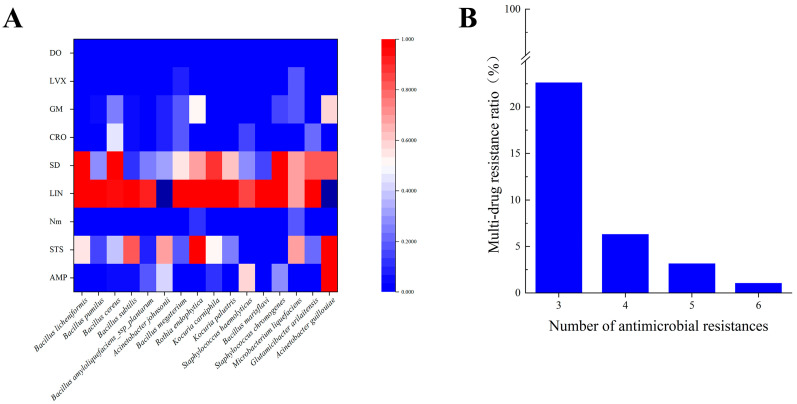
Antimicrobial resistance of the isolates. (**A**) Susceptibility of various bacteria to different antimicrobials. The antimicrobial resistance rates were analyzed for isolates with ≥5 isolates (16 species, 190 isolates). The susceptibility of different isolates to nine antimicrobials varied, with the mean resistance rate of LIN being 87.37% (166/190), SD 61.05% (116/190), STS 42.63% (81/190), and the DO being 0%. (**B**) Multidrug resistance of bacteria. Three-drug resistance was the most common among MDR isolates at 22.63% (43/190), most MDR isolates were resistant to three–four antimicrobials, accounting for 87.3% (55/63) of MDR isolates, and the most resistant MDR isolates were resistant to six antimicrobials at the same time.

In this study, a total of nine common antimicrobial agents were screened for antimicrobial resistance and the minimum inhibitory concentration (MIC) of 276 isolates was determined. The MIC of isolates with too few numbers would have some uncertainty in regard to use as credible data, so we only analyzed the resistance rate for isolates with ≥5 isolates (16 species, 190 isolates). The results showed that the susceptibility of all isolates to the nine antimicrobials varied, and the overall resistance rate ranged from 0% to 87.37%, with a mean resistance rate of 87.37% (166/190) for LIN, 61.05% (116/190) for SD, 42.63% (81/190) for STS, and 0% for DO, which had the lowest resistance rate (Figure 4A). The MDR rate of the bacteria isolated in this experiment was relatively low, only 33.16% (63/190) compared to the 100% found by Li et al. [12]. Resistance to three antimicrobials was the most prevalent among MDR isolates at 22.63% (43/190). Most MDR isolates were resistant to three–four antimicrobials, accounting for 87.3% (55/63) of multi-resistant isolates. The most antimicrobial-resistant MDR isolates were resistant to six antimicrobials simultaneously (Figure 4B).

### 3.3. Antimicrobial Resistance Genes

**Figure 5:**
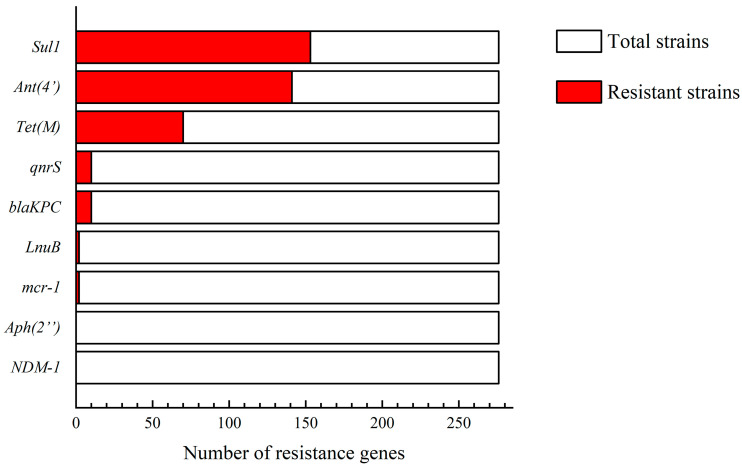
Antimicrobial resistance gene testing. Nine antimicrobial resistance genes were tested in 276 bacterial isolates, and a total of seven of these antimicrobial resistance genes were identified. The highest positive rate was for *Sul*1 (55.43%), the lowest was for *mcr-1* and *LnuB* (Both are 0.72%), and the resistance genes NDM-1 and *aph(2″)* were not detected.

In the current study, nine antimicrobial resistance genes were tested in 276 bacterial isolates, with seven of them having positive rates ranging from 55.43% to 0.72%. The highest positive rate was *Sul*1 (55.43%), the lowest was *mcr-1* and *LnuB* (0.72%), and neither NDM-1 nor *aph(2″)* was detected (Figure 5). Taken together, the positive rate of antimicrobial resistance genes in dairy farms in Shandong Province was generally lower than that in broiler farms compared to the antimicrobial resistance genes detected in broiler farms in Shandong Province by Li et al. (94% *Sul*1 detection rate) [12].

## 4. Discussion

This study reported that the bacterial positivity rate of teat cup liners after milking in Shandong was 61.69% (153/248), indicating that more than half of the raw milk may be contaminated with bacteria from the udder surface or teat cup lining during milking, which reminds us that disinfection of cow udders and milking equipment may not be taken very seriously in actual production. Notably, among these isolated bacteria, *B. cereus* can cause milk spoilage, wound infections, and systemic diseases [13]. The capacity of Bacillus to generate biofilms and spores under adverse circumstances significantly reduces the disinfecting impact and introduces a notable level of resistance to the pasteurization procedures utilized in food processing. As a result, once food is contaminated by Bacillus, complete disinfection becomes arduous to achieve [14]. Tens of thousands of food poisoning incidents occur worldwide each year, and it is reported that 1.4–12% of them are caused by *Bacillus cereus* (the real number may be higher due to incomplete statistics) [15]. The European Food Safety Authority 2018 annual report shows that “bacterial toxins other than *Clostridium botulinum*” (including *Bacillus cereus*) typically account for 16–20% of food poisoning incidents, second only to *salmonella* and viruses [16]. *B. cereus* is one of the major microbial factors limiting the shelf life of pasteurized milk, with 30% of pasteurized milk samples from Poland reported to have detected *Bacillus cereus*, in addition to 37% from India and 27% from Chinese samples [17,18]. It was also reported that the isolation rate of *B. cereus* in domestic pasteurized milk was 41.23%, indicating that *B. cereus* contamination is a common problem in pasteurized dairy products [19]. In this study, the total detection rate of *B. cereus* was 6.16% (17/276), which is lower than the above study. The higher detection rate of Bacillus in pasteurized milk compared to our findings may be attributed to two potential factors. Firstly, the bulk mixing of milk from multiple sources could have contributed to the increased prevalence of Bacillus spores in the samples. Secondly, the resistance of Bacillus spores to the pasteurization process may have enabled their survival, leading to the higher detection rates observed in the pasteurized milk. 

Over time, antimicrobial resistance in bacteria has become increasingly serious. The antimicrobial susceptibility test in our study showed that the isolated bacteria were resistant to eight of the nine antimicrobials to varying degrees. The highest rate of resistance to LIN (87.37%) and the lowest rate of resistance to DO (0%) were observed. Resistance rates to SD and STS also remained at high levels (more than or close to 50%). A report showed that bacteria isolated from raw milk in Henan Province (China) had 95.50% resistance to lincomycin, which is very close to our findings [20]. This study also found that the MDR of bacteria was a serious phenomenon, with MDR isolates accounting for 33.16% (63/190) of the total number of isolates, and most MDR isolates were resistant to three to four antimicrobials, accounting for 87.3% (55/63) of multidrug-resistant isolates. The most resistant MDR isolates were resistant to six antimicrobials simultaneously. However, our MDR detection rate is still relatively low compared to the 43.93% reported in Hubei, China, [21] and the 96.4% reported in Ethiopia [22]. Likewise, the level of antimicrobials in the environment of dairy farms in South China has been tested, and antimicrobial residues were found in manure, wastewater, and suspended particles [23]. As the isolated bacteria are usually highly resistant to LIN, SD, and STS, dairy farms in Shandong Province should minimize or avoid the use of these three antimicrobial agents and pay attention to the rotation and combination of antimicrobial agents to minimize the emergence of antimicrobial-resistant strains.

The prevalence of antimicrobial-resistant bacteria and antimicrobial resistance genes (ARGs) is a major public health concern for people worldwide, and an important factor in bacteria exhibiting antimicrobial resistance is that they carry an associated antimicrobial resistance gene. Happily, the feared NDM-1 resistance gene was not detected, and the detection rate of *mcr-1* was very low. Our data showed that the detection rate of the sulfonamide resistance gene *Sul*1 was as high as 55.43%, followed by the aminoglycoside resistance gene *ant*(4′) (51.09%), the tetracycline resistance gene *tet*(M) (25.36%), the β-lactam resistance gene *bla*_KPC_ (3.62%), the quinolone resistance gene *qnrS* (3.62%), the polymyxin resistance gene *mcr-1* (0.72%) and the lincosamide resistance gene *LnuB* (0.72%); the β-lactam resistance gene *NDM-1* and the aminoglycoside resistance gene *Aph*(2″) were not detected. Statistical analysis of the results revealed a positive trend of correlation between some resistance genes and resistance phenotypes, such as sulfonamides. However, it was also found that there was a discrepancy between antimicrobial resistance genes and antimicrobial resistance phenotypes; for example, the resistance rate of tetracycline was 0, but the detection rate of *tet*(M), a resistance gene of tetracyclines, was 25.36%. To seek an answer to this question, we reviewed the relevant literature and found that this may be because most acquired ARGs need to be overexpressed before conferring resistance, and this overexpression also needs to meet certain conditions [24]. Therefore, we thought that this inconsistency might be due to this reason. On the other hand, the average resistance rate of lincomycin was 87.37%, but the detection rate of *LnuB* was only 0.72%, which may be due to the presence of other types of resistance genes leading to the expression of resistance, such as *LnuC*, *LnuG*, and *LnuH* [25,26,27].

## 5. Conclusions

This study reported that post-milking teat cup liners from dairy farms in Shandong Province had a high bacterial isolation rate (>50%) and an abundance of bacterial species. The presence of *B. cereus*, which can cause milk spoilage and food poisoning, a situation that poses a potential threat to food safety and public health, was also detected. The overall antimicrobial resistance profile had an alarming average resistance rate of 87.37% for lincomycin and over 50% for sulfonamides, with most isolates being insusceptible to multiple antimicrobials. Nine antimicrobial resistance genes were examined, and the highest detection rate of 55.43% was found for the sulfa resistance gene *Sul*1. In addition, the polymyxin resistance gene *mcr-1* was isolated from bacteria in the cup liner for the first time. These results have a reference value for the clinical use of antimicrobials in dairy farms in Shandong Province and provide valuable information on the potential transmission of antimicrobial resistance genes through the food chain.

## Figures and Tables

**Table 1:** Percentage of samples positive for bacterial isolation (number of tests/total number).

Region	Farm 1	Farm 2	Total
Tai’an	39.47% (30/76)	63.46% (33/52)	49.22% (63/128)
Dongying	63.33% (19/30)	73.33% (22/30)	68.33% (41/60)
Qingdao	100% (30/30)	63.33% (19/30)	81.67% (49/60)
Total	-	-	61.69% (153/248)

## Data Availability

The raw data supporting the conclusions of this article will be made available by the authors on request.

## Supplementary Materials

The following supporting information can be downloaded at: https://www.mdpi.com/article/10.3390/ani14152167/s1, Table S1: PCR primers used in the study [28,29,30,31,32].

## References

[1] Smith N.W., Fletcher A.J., Hill J.P., McNabb W.C. (2021). Modeling the Contribution of Milk to Global Nutrition. Front. Nutr..

[2] Liu X.-Y., Hu Q., Xu F., Ding S.-Y., Zhu K. (2020). Characterization of *Bacillus cereus* in dairy products in China. Toxins.

[3] Antunes P., Novais C., Peixe L. (2020). Food-to-Humans Bacterial Transmission. Microbiol. Spectr..

[4] Duse A., Persson-Waller K., Pedersen K. (2021). Microbial aetiology, antibiotic susceptibility and pathogen-specific risk factors for udder pathogens from clinical mastitis in dairy cows. Animals.

[5] Mola I., Onibokun A., Oranusi S. (2021). Prevalence of multi-drug resistant bacteria associated with foods and drinks in Nigeria (2015–2020): A systematic review. Ital. J. Food Saf..

[6] Khan A.U., Maryam L., Zarrilli R. (2017). Structure, Genetics and Worldwide Spread of New Delhi Metallo-beta-lactamase (NDM): A threat to public health. BMC Microbiol..

[7] Yong D., Toleman M.A., Giske C.G., Cho H.S., Sundman K., Lee K., Walsh T.R. (2009). Characterization of a new metallo-beta-lactamase gene, bla(NDM-1), and a novel erythromycin esterase gene carried on a unique genetic structure in Klebsiella pneumoniae sequence type 14 from India. Antimicrob. Agents Chemother..

[8] Liu Y.Y., Wang Y., Walsh T.R., Yi L.X., Zhang R., Spencer J., Doi Y., Tian G., Dong B., Huang X. (2016). Emergence of plasmid-mediated colistin resistance mechanism MCR-1 in animals and human beings in China: A microbiological and molecular biological study. Lancet Infect Dis..

[9] Bell B.G., Schellevis F., Stobberingh E., Goossens H., Pringle M. (2014). A systematic review and meta-analysis of the effects of antibiotic consumption on antibiotic resistance. BMC Infect Dis..

[10] Junior J.C.R., Tamanini R., Soares B.F., de Oliveira A.M., de Godoi Silva F., da Silva F.F., Augusto N.A., Beloti V. (2016). Efficiency of boiling and four other methods for genomic DNA extraction of deteriorating spore-forming bacteria from milk. Semin. Ciênc. Agrár..

[11] Stenfors Arnesen L.P., Fagerlund A., Granum P.E. (2008). From soil to gut: *Bacillus cereus* and its food poisoning toxins. FEMS Microbiol. Rev..

[12] Li Z., Peng C., Zhang G., Shen Y., Zhang Y., Liu C., Liu M., Wang F. (2022). Prevalence and characteristics of multidrug-resistant Proteus mirabilis from broiler farms in Shandong Province, China. Poult. Sci..

[13] Ehling-Schulz M., Lereclus D., Koehler T.M. (2019). The *Bacillus cereus* Group: *Bacillus* Species with Pathogenic Potential. Microbiol. Spectr..

[14] Martlbauer E., Granum P.E. (2021). *Bacillus cereus* Toxins. Toxins.

[15] Grutsch A.A., Nimmer P.S., Pittsley R.H., Kornilow K.G., McKillip J.L. (2018). Molecular pathogenesis of *Bacillus* spp., with emphasis on the dairy industry. Fine Focus.

[16] European Food Safety Authority, European Centre for Disease Prevention and Control (2018). The European Union summary report on trends and sources of zoonoses, zoonotic agents and food-borne outbreaks in 2017. EFSA J..

[17] Berthold-Pluta A., Pluta A., Garbowska M., Stefanska I. (2019). Prevalence and toxicity characterization of *Bacillus cereus* in food products from Poland. Foods.

[18] Gao T., Ding Y., Wu Q., Wang J., Zhang J., Yu S., Yu P., Liu C., Kong L., Feng Z. (2018). Prevalence, Virulence Genes, Antimicrobial Susceptibility, and Genetic Diversity of *Bacillus cereus* Isolated From Pasteurized Milk in China. Front. Microbiol..

[19] Zhai Z., Cui C., Li X., Yan J., Sun E., Wang C., Guo H., Hao Y. (2023). Prevalence, antimicrobial susceptibility, and antibiotic resistance gene transfer of *Bacillus* strains isolated from pasteurized milk. J. Dairy Sci..

[20] Liu B.G., Xie M., Gong Y.T., Dong Y., Zheng G.M., Wu H., Hu G.Z., Bai M., Xu E.P. (2022). Prevalence, resistance phenotypes, and fluoroquinolone resistance genes of Salmonella isolates from raw milk of healthy dairy cows in Henan province, China. Eur. Rev. Med. Pharmacol. Sci..

[21] Wu X., Liu J., Feng J., Shabbir M.A.B., Feng Y., Guo R., Zhou M., Hou S., Wang G., Hao H. (2022). Epidemiology, Environmental Risks, Virulence, and Resistance Determinants of *Klebsiella pneumoniae* from Dairy Cows in Hubei, China. Front. Microbiol..

[22] Abunna F., Ashenafi D., Beyene T., Ayana D., Mamo B., Duguma R. (2017). Isolation, identification and antimicrobial susceptibility profiles of Salmonella isolates from dairy farms in and around Modjo town, Ethiopia. Ethiop. Vet. J..

[23] Zhou L.J., Ying G.G., Liu S., Zhang R.Q., Lai H.J., Chen Z.F., Pan C.G. (2013). Excretion masses and environmental occurrence of antibiotics in typical swine and dairy cattle farms in China. Sci. Total Environ..

[24] Nielsen T.K., Browne P.D., Hansen L.H. (2022). Antibiotic resistance genes are differentially mobilized according to resistance mechanism. Gigascience.

[25] Achard A., Villers C., Pichereau V., Leclercq R. (2005). New lnu(C) gene conferring resistance to lincomycin by nucleotidylation in *Streptococcus agalactiae* UCN36. Antimicrob. Agents Chemother..

[26] Li Y., Qiu Y., She J., Wang X., Dai X., Zhang L. (2021). Genomic Characterization of a *Proteus* sp. Strain of Animal Origin Co-Carrying bla(NDM-1) and lnu(G). Antibiotics.

[27] Luo H.Y., Liu M.F., Wang M.S., Zhao X.X., Jia R.Y., Chen S., Sun K.F., Yang Q., Wu Y., Chen X.Y. (2018). A novel resistance gene, lnu(H), conferring resistance to lincosamides in *Riemerella anatipestifer* CH-2. Int. J. Antimicrob. Agents.

[28] Stepien-Pysniak D., Hauschild T., Dec M., Marek A., Brzeski M., Kosikowska U. (2021). Antimicrobial resistance and genetic diversity of Enterococcus faecalis from yolk sac infections in broiler chicks. Poult. Sci..

[29] Poirel L., Walsh T.R., Cuvillier V., Nordmann P. (2011). Multiplex PCR for detection of acquired carbapenemase genes. Diagn. Microbiol. Infect. Dis..

[30] Li R., Xie M., Zhang J., Yang Z., Liu L., Liu X., Zheng Z., Chan E.W., Chen S. (2017). Genetic characterization of mcr-1-bearing plasmids to depict molecular mechanisms underlying dissemination of the colistin resistance determinant. J. Antimicrob. Chemother..

[31] Doma A.O., Popescu R., Mituletu M., Muntean D., Degi J., Boldea M.V., Radulov I., Dumitrescu E., Muselin F., Puvaca N. (2020). Comparative Evaluation of qnrA, qnrB, and qnrS Genes in Enterobacteriaceae Ciprofloxacin-Resistant Cases, in Swine Units and a Hospital from Western Romania. Antibiotics.

[32] Chaturvedi P., Singh A., Chowdhary P., Pandey A., Gupta P. (2021). Occurrence of emerging sulfonamide resistance (sul1 and sul2) associated with mobile integrons-integrase (intI1 and intI2) in riverine systems. Sci. Total Environ..

