# Supplementary material for: Bacterial Diversity and Antimicrobial Resistance of Microorganisms Isolated from Teat Cup Liners in Dairy Farms in Shandong Province, China
Source: Animals (Basel). 2024 Jul 25;14(15):2167. doi: 10.3390/ani14152167 (PMC11311105; doi:10.3390/ani14152167)
Supplement: Supplementary file 1 [file animals-14-02167-s001.zip › animals-3077785-SI.pdf]

Table S1. PCR primers used in the study

Gene	Sequence (5' to 3')	Amplicon size (bp)	Reference
mcr-1	F: CGGTCAGTCCGTTTGTTC<br>R: CTTGGTCGGTCTGTAGGG	309	(Li *et al.*, 2017)
NDM-1	F: GTCTGGCAGCACACTTCCTATCTC<br>R: GGTTCGACAACGCATTGGCATAAG	268	-
bla $_{KPC}$	F: CGTCTAGTTCTGCTGTCTTG<br>R: CTTGTCATCCTTGTTAGGCG	798<br>232	(Poirel *et al.*, 2011)
qnrS	F: ACGACATTCGTCAACTGGAA<br>R: TTAATTGGCACCCTGTAGGC	417	(Doma *et al.*, 2020)
sul1	F: TTCGGCATTCTGAATCTCAC<br>R: ATGATCTAACCCTCGGTCTC	822	(Chaturvedi *et al.*, 2021)
aph(2'')	F: CCACAATGATAATGACTCAGTTCCC<br>R: CCACAGCTTCCGATAGCAAGAG	444	(Stepien-Pysniak *et al.*, 2021)
ant(4')	F: CAAACTGCTAAATCGGTAGAAGCC<br>R: GGAAAGTTGACCAGACATTACGAACT	294	(Stepien-Pysniak *et al.*, 2021)
tet(M)	F: GTGGACAAAGGTACAACGAG<br>R: CGGTAAAGTTCGTCACACAC	406	(Stepien-Pysniak *et al.*, 2021)
lnuB	F: CCTACCTATTGTTTGTGGAA<br>R: ATAACGTTACTCTCCTATTC	925	(Stepien-Pysniak *et al.*, 2021)
